# Acute dynamic conduction disorder after transcatheter aortic valve implantation

**DOI:** 10.1093/ehjcr/ytae089

**Published:** 2024-02-14

**Authors:** Jesus Ricardo Pérez-Castellanos, Ivanhoe Arellano-Bernal, Elias Noel Andrade-Cuellar

**Affiliations:** Department of Cardiology, National Medical Center November 20, Av. Felix Cuevas #540, Col. Del Valle Del. Benito Juarez, Mexico City 03100, Mexico; Department of Cardiology, National Medical Center November 20, Av. Felix Cuevas #540, Col. Del Valle Del. Benito Juarez, Mexico City 03100, Mexico; Department of Cardiology, National Medical Center November 20, Av. Felix Cuevas #540, Col. Del Valle Del. Benito Juarez, Mexico City 03100, Mexico

## Case

A 77-year-old woman with a history of hypothyroidism, arterial hypertension, diabetes, and chronic renal disease underwent evaluation for a 6-month history of dyspnoea and chest pain. Transthoracic echocardiogram revealed severe symptomatic aortic stenosis classified as American Heart Association/American College of Cardiology D1. Transcatheter aortic valve replacement (TAVR) was scheduled due to her high surgical risk.

**Figure 1:**
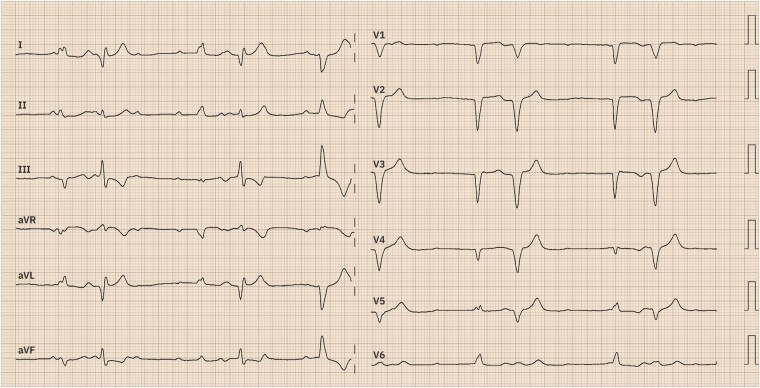
Electrocardiogram.

Shortly after the deployment of a self-expandable valve, she experienced sudden nausea and dyspnoea, with these symptoms being associated with progressive hypotension. Electrocardiographic images were captured as depicted below (*Figure 1*).

## Question 1

### What is the electrocardiographic diagnosis?

- New left bundle branch block (LBBB)
- Right bundle branch block (RBBB) with left anterior bundle branch block (LABBB)
- RBBB with left posterior bundle branch block (LPBBB)
- Trifascicular block with complete atrioventricular (AV) block
- Alternating bundle branch block

The correct answer is D.

## Discussion and explanation

A trifascicular block can manifest in two distinct patterns: either as an alternating complete LBBB and RBBB or as a combination of RBBB with alternating LABBB and LPBBB.^1,2^ It is important to highlight that the combination of bifascicular block (RBBB plus LABBB, RBBB plus LPBBB, or LBBB) with first-degree AV block cannot be considered as trifascicular block because the site of AV conduction delay can reside in either the AV node or His–Purkinje system; hence, such pattern can reflect slow conduction in the AV node with concomitant bifascicular block rather than disease in the third fascicle.^3^

Subsequent to the deployment of the self-expandable valve (see Supplementary material online, *Video S1*), the patient presented symptoms indicative of low cardiac output, including hypotension. This was accompanied by the onset of a new LBBB, which subsequently alternated between RBBB and LPBBB, as illustrated in Supplementary material online, *Figure S1*. The condition progressed to culminate in a complete AV block, as depicted in Supplementary material online, *Figure S2*.

## Question 2

### What is the anatomical substrate explaining the appearance of these electrical alterations after TAVR?

- Large membranous septum
- The close relationship between the LBBB and the base of the interleaflet triangle separating the non-coronary and right coronary leaflets of the aortic valve
- The close relationship between the RBBB and the base of the non-coronary leaflet of the aortic valve
- The close relationship of the bundle branch with the base of the interleaflet triangle separating the right and left coronary leaflets of the aortic valve
- The anterosuperior relationship of the AV node with the apex of the Koch triangle

The correct answer is B.

## Discussion and explanation

The proximity of the conduction system to the aortic valve elucidates the genesis of electrical disturbances post-TAVR due to local insults like oedema, ischaemia, and haematoma. The AV node has an anteroposterior relationship to the apex of the Koch triangle; hence, Answer E is incorrect. The correct answer is B, as the AV node continues as the bundle of His, piercing the membranous septum and penetrating to the left, exiting just beneath the membranous septum and superficially positioning itself on the interventricular septum. Here, the left bundle branch is intimately related to the base of the interleaflet triangle separating the non-coronary and right coronary leaflets of the aortic valve,^3^ thereby excluding the incorrect anatomical relationships of Answers C and D.

## Question 3

### What is the recommendation for cardiac stimulation for this conduction disorder?

- Permanent pacemaker for new LBBB with QRS > 150ms
- Permanent pacemaker for PR prolongation > 240ms
- Permanent pacemaker for complete AV block
- Temporary pacemaker for 48 h due to a new alternating BBB
- Does not require a permanent pacemaker; the next step is electrographic monitoring for 48 h.

The correct answer is C.

## Discussion and explanation

Electrocardiographic monitoring for 48 h is only indicated when the patient presents a new LBBB with QRS prolongation > 150 ms and first-degree AV block with PR prolongation > 240 ms, so Options A, B, and E are incorrect. In patients undergoing TAVR, the presence of alternating BBB and complete or high-grade AV block are indications for permanent pacemaker implantation, making Option C the correct choice.^4^ The patient presented with a complete AV block associated with a trifascicular block immediately after valve implantation, necessitating permanent stimulation with a dual-chamber pacemaker in DDD mode (see Supplementary material online, *Figure S3*).

## Supplementary Material

ytae089_Supplementary_Data

## Data Availability

The data underlying this article are available in the article and in its online Supplementary material.
