# Supplementary figures and images for: Acute dynamic conduction disorder after transcatheter aortic valve implantation
Source: Eur Heart J Case Rep. 2024 Feb 14;8(2):ytae089. doi: 10.1093/ehjcr/ytae089 (PMC10893999; doi:10.1093/ehjcr/ytae089)

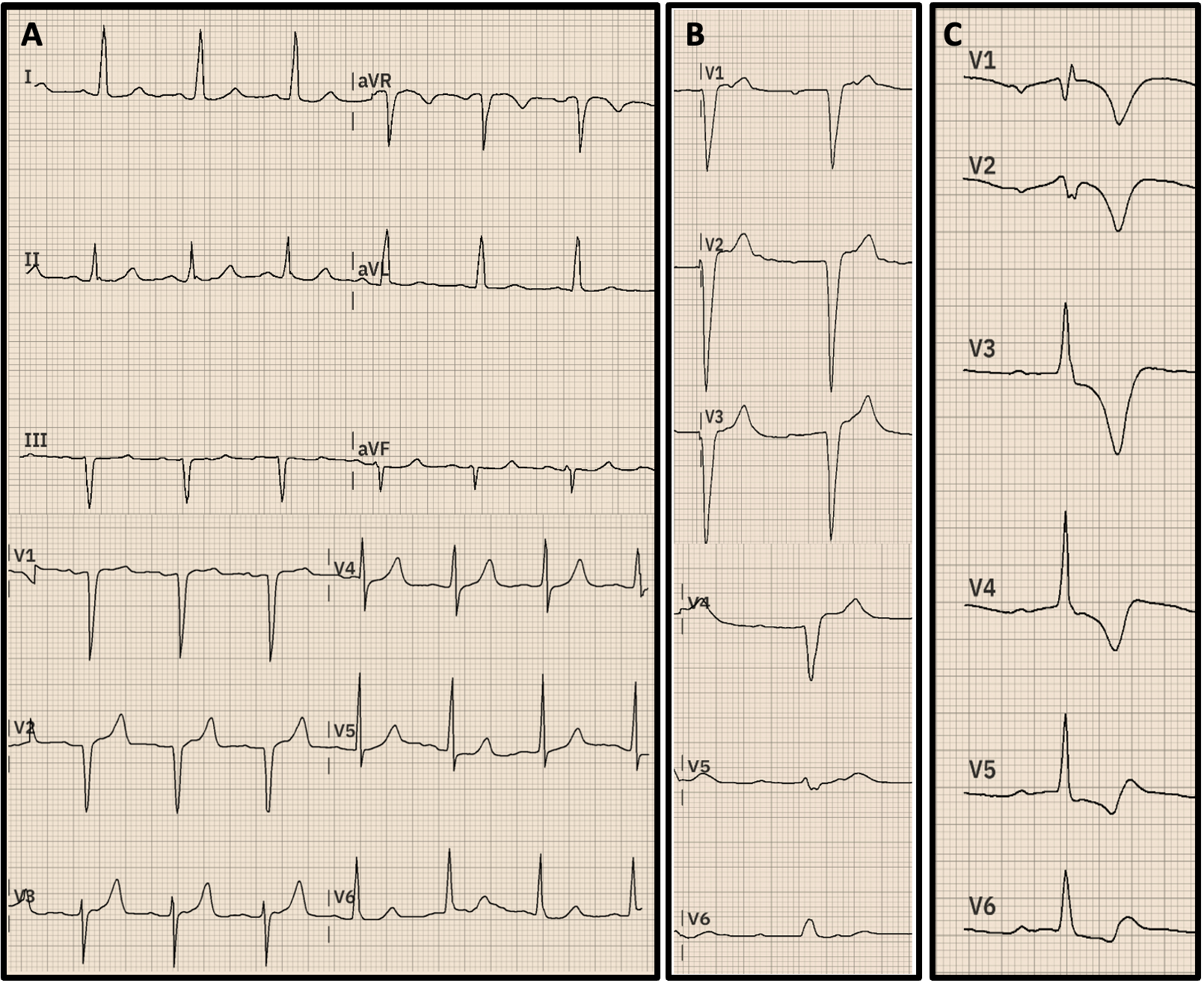

Supplement: ytae089_Supplementary_Data [file ytae089_supplementary_data.zip › Figure S1.jpg]

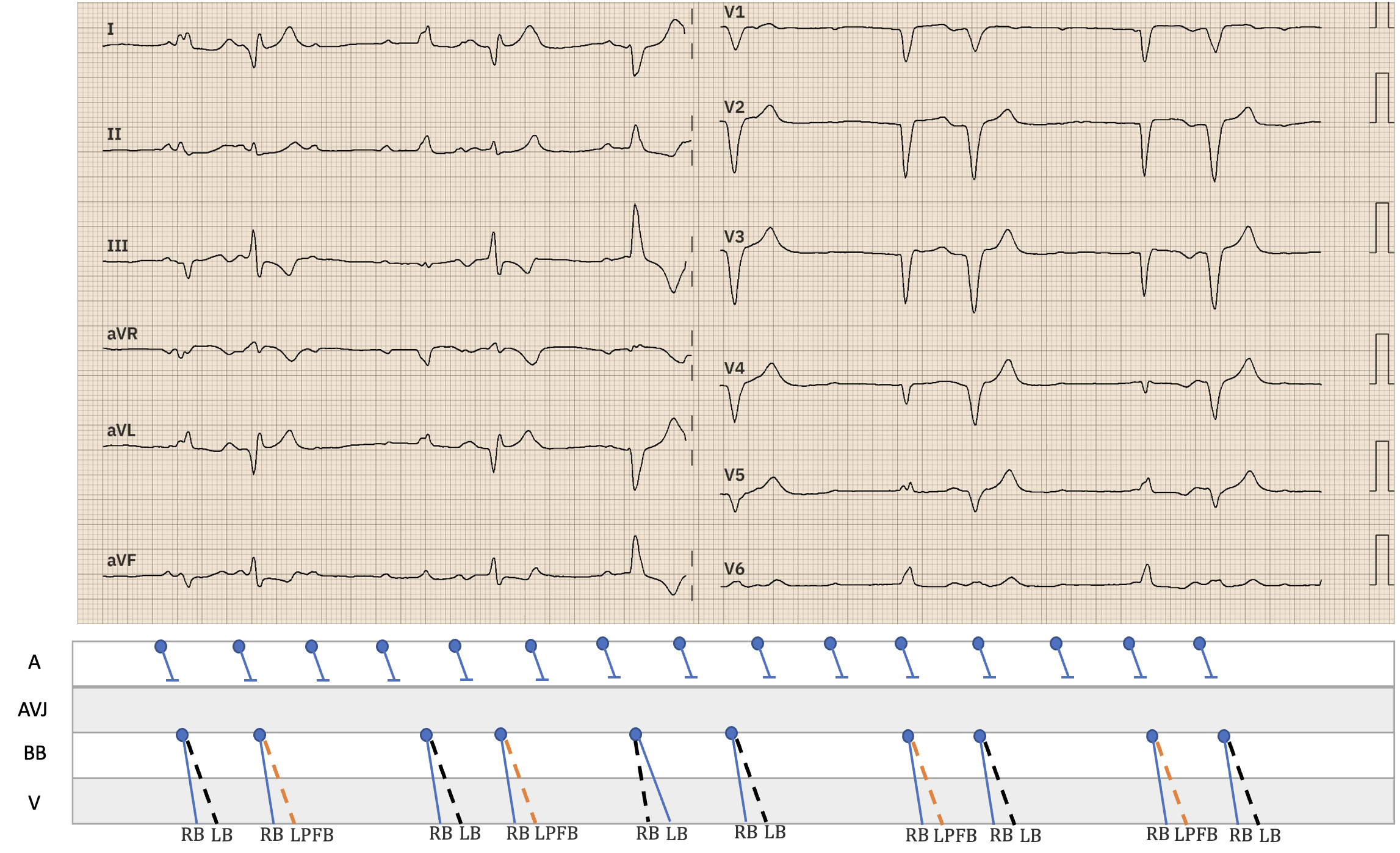

Supplement: ytae089_Supplementary_Data [file ytae089_supplementary_data.zip › Figure S2.jpg]

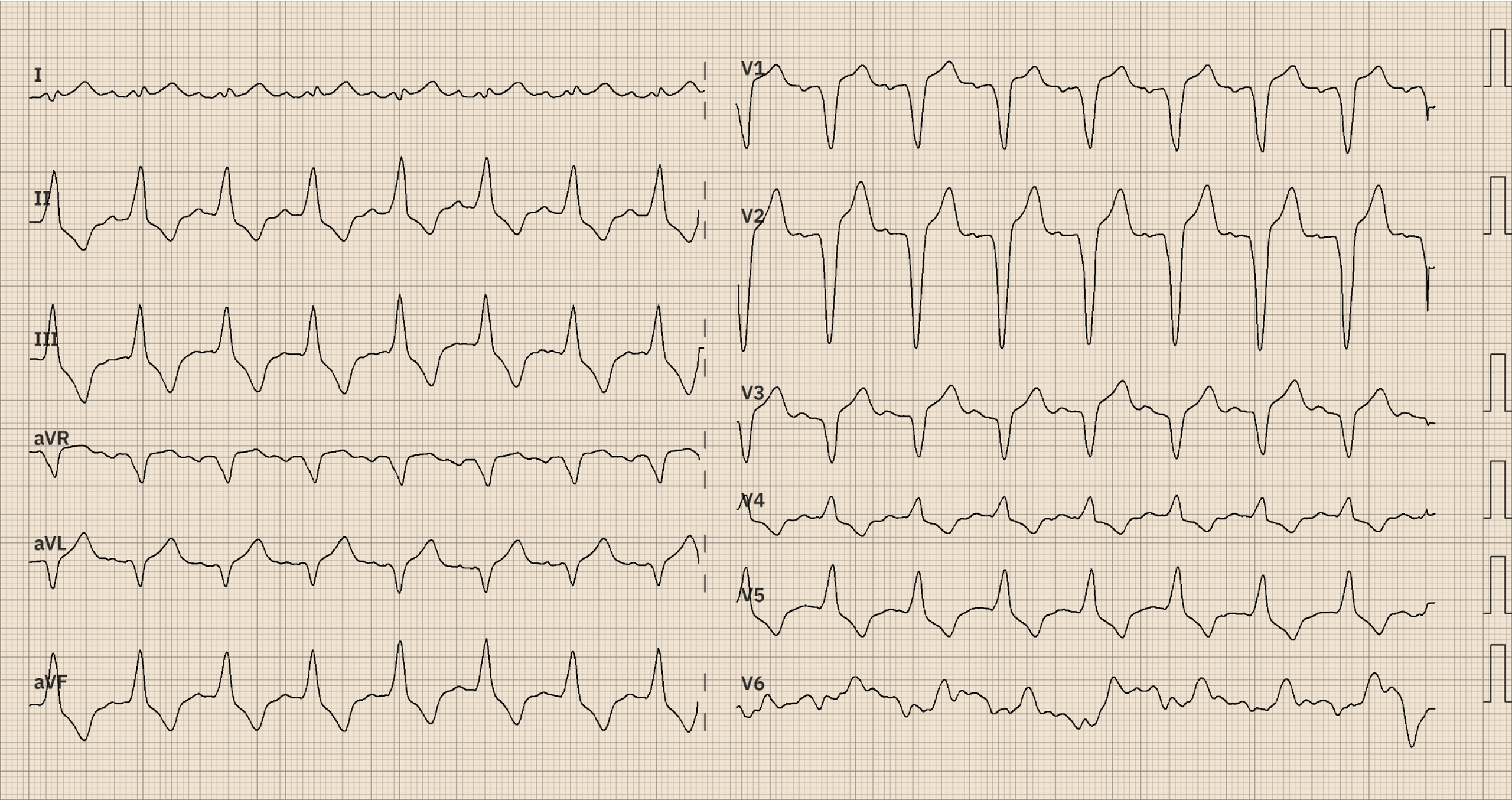

Supplement: ytae089_Supplementary_Data [file ytae089_supplementary_data.zip › Figure S3.jpg]
